# A Multipurpose Platform for Ambient Assisted Living (ActiveAdvice): Usability Study

**DOI:** 10.2196/18164

**Published:** 2021-03-01

**Authors:** Diogo Abrantes, Soraia Teles, Rita Tavares de Sousa, Alberto Freitas, Pedro Vieira-Marques, Ana Ferreira

**Affiliations:** 1 Center for Health Technology and Services Research University of Porto Porto Portugal; 2 Department of Behavioral Sciences Institute of Biomedical Sciences Abel Salazar University of Porto Porto Portugal

**Keywords:** aging, ambient assisted living, elderly, usability testing, user-centered design

## Abstract

**Background:**

Aging of the global population is slowly paving the way for new markets for care products and services. The desire of older people to maintain their independence while remaining at home is boosting the development of ambient assisted living (AAL) solutions. Lack of user awareness of AAL solutions paired with an insufficient use of user-centered and participatory design approaches in the development of these products has hindered the uptake of these solutions by end users.

**Objective:**

This study aims to describe the usability and users’ experiences within a novel platform, ActiveAdvice, aimed at offering advice and a holistic market overview of AAL products and services.

**Methods:**

Usability tests were performed on the developed platform among identified prospective end users, with 32 older adults and informal carers from 4 European countries being part of the user tests. The usability and appeal of the web interface design, information flow, and information architecture were analyzed by collecting both objective and subjective measures. These would include pretest and posttest surveys, along with a series of think-aloud tasks to be performed within the platform.

**Results:**

The outcomes suggest that the ActiveAdvice platform’s objectives and functionalities are mostly aligned with the needs and expectations of end users, who demonstrated interest in using it, stressing its purpose along with its simple and intuitive interaction. Task completion rates were high, and participants had good satisfaction rates when navigating the platform. However, the tests still advocate for an improved design at some points and better disclosure of information.

**Conclusions:**

Our findings shed light on a few peculiarities of interface design, information architecture, user needs, and preferred functionalities, which should be applied to future developments of similar platforms with related services. The AAL field could benefit from tools supporting the dissemination of available AAL solutions and how they can improve one's quality of life. These tools may benefit not only older adults but also caregivers, business owners, and governmental employees.

## Introduction

### Background

The world’s population is aging. Several countries are now experiencing a demographic shift, which translates into a rising proportion of older people among their inhabitants. According to data from the United Nations’ World Population Prospects [1], the number of older persons (aged 60 years or older) is expected to more than double by 2050 and more than triple by 2100, rising from 962 million globally in 2017 to 2.1 billion in 2050 and 3.1 billion in 2100. We should also consider a decrease in physical and mental abilities and the impact of age-related or chronic diseases such as Alzheimer disease and Parkinson disease. In response, new markets for care products and services are aiming to provide older people with a higher level of autonomy and quality of life [2]. As reported by a number of studies [3-5], older people would prefer to spend time in their home or a familiar environment, prioritizing their independence rather than being taken care of, especially in institutional settings. A variety of new possibilities allowing older adults to retain a degree of autonomy at home is now being offered by the progressive use of information and communication technologies, which can also help fend off issues such as isolation and loneliness, both linked to physical and mental decline [6]. These technologies are based on the ambient intelligence paradigm, along with the concept of ambient assisted living (AAL), and address the struggles that arise from this demographic shift [7,8]. The development of most AAL systems is based on the implementation of pervasive and unobtrusive devices, which is meant to increase autonomy and quality of life [9]. These AAL tools can assist in a variety of ways and can be divided into 3 categories, in line with people’s needs as they grow older: devices for everyday activities, home safety equipment, and technology for social participation [10-12].

Researchers in the field of aging and human factors have been investigating a number of pre- and postimplementation elements that can hinder the adoption of and influence the attitudes toward technology for aging in place. The barriers were identified as the characteristics of older persons (perceived needs, technological skills, and medical conditions), their environment (social support for technology use, living environment), and technology features (hardware, interface design, usability testing, and accessibility) [13-16]. The design of systems that are intended to be used by older people is often highly technology-oriented instead of user-oriented, being mostly defined from the ground up by the analysis of available technologies rather than by the users’ needs. The image the system delivers and the mental models that come with it should be carefully studied by designers to avoid producing something that presents the older users with a metaphor they do not understand at a fundamental level [17,18]. As the need to pay special attention to user research and usability testing was recognized, better approaches for conceiving new technological developments came to be in demand. Getting the end users involved has been shown to be among the most successful strategies in fomenting engagement and trust with those technologies [19]. It is widely agreed that both user-centered design (UCD) and participatory design (PD) are meaningful approaches when designing AAL solutions, and their importance is shown in a variety of different studies, despite failing to prevail in technological developments [18,20,21]. This issue also seems to contribute to a well-identified challenge in the uptake of AAL solutions by end users [18,22,23], paired with the low level of general public awareness of AAL solutions [22-24].

Efforts to raise awareness among potential users of AAL technologies are currently undertaken by publishing informative websites on the internet, but they often focus on comprehensive information for older adults on topics such as nutrition, leisure and sports, and events, falling short in dedicating a bigger part to AAL. When the topic is mentioned, the most commonly presented information only explains the AAL concept and its implications, hardly delivering any comprehensive and well-structured overview of existing technologies or solution providers. There are no thoroughly tested, trustworthy, reliable, and established platforms that gather information about AAL and related products in a clear and understandable way, while providing options to know more or acquire them online. Although existing websites [25,26] could be considered as projects that started to fill the gap on the matter, they lack particular key elements deemed necessary to solidify and disseminate the concept. Building an online product catalog without tending to and validating other issues such as feasibility, functionality, or usability can be proven unproductive or fruitless, hence the scope to improve.

The ActiveAdvice [27] project, a European AAL-funded project carried out in 6 countries (Austria, Belgium, the Netherlands, Portugal, Switzerland, and the United Kingdom), was developed to address these gaps by delivering a web platform directed to older adults and their relatives, AAL business representatives, as well as governmental organizations involved in aging issues across Europe. The platform offers a holistic market overview, presenting a directory of AAL products and services while combining it with a group of advisory functionalities that can inform and guide users in the process of finding a product suited to their needs.

This study aims to evaluate the level of interest, feasibility, and usability of the ActiveAdvice platform among its prospective primary and secondary end users. Primary end users are, as defined by the AAL program [28], individuals using a product or service for a direct benefit of their quality of life—here, the older adults—while secondary end users—here, the informal carers (ICs)—are individuals who may benefit from products and services indirectly providing the reduction of primary end users’ care needs. Informal caregivers may also enable older adults to search for and use AAL solutions. We recruited older adults and informal caregivers to enroll in a number of test sessions to test a prototype of the ActiveAdvice platform and presented and discussed the findings and takeaways produced from a variety of challenges. To frame the prototype under study, the next section presents the development process and outcomes of the ActiveAdvice platform. The following sections describe the usability study, along with a presentation and discussion of the results. All study procedures were approved by the ethics committee of the University Hospital Center of S. João/Faculty of Medicine of the University of Porto (CE-305-2020).

### The ActiveAdvice Platform

The ActiveAdvice platform originated from a European Union or AAL program–funded project intended to set up a European-wide advisory and decision support platform that brings together a broad range of available AAL products, services, and experts. As stressed by Nedopil et al [29], although AAL projects are substantially diverse, they all share a basic innovation process consisting of 3 basic stages: (1) understanding the users, including their characteristics, necessities, and requirements; (2) conceptualizing the solution, namely use cases, technology elements, and implications for users; and (3) testing the full solution or parts, as well as its benefits. Although this paper delves into the third phase (testing), we briefly discuss the previous stages, with a greater emphasis on the conceptualized solution to better frame the testing procedures.

### Understanding the Users: Requirement Analysis

A user-centered requirements engineering methodology puts the intended user at the center. Stakeholder needs, interests, and expectations need to be transferred into requirements and subsequently into measurable qualities, assisting the creation of a better layout of the platforms’ representation and functionality. It also helps create a common vision for the developers, free of implicit assumptions and technological constraints. For the ActiveAdvice platform development, the integration of stakeholders at a very early stage of the project was a precondition, and thus, several stakeholder groups were integrated in a requirement analysis stage. This allowed for a better understanding of their perspectives, insights, motivations, and concerns, clarifying what could help, as well as hinder the development and implementation of the ActiveAdvice platform. The process featured a total of 38 semistructured interviews with stakeholders (12 end users, 14 business representatives in the AAL market, and 12 government representatives engaged in aging issues) in 5 European countries (Austria, Belgium, the Netherlands, Portugal, Switzerland, and the United Kingdom), with the results fully described by Teles et al [30], supporting the subsequent development of the platform.

### Conceptualizing the Solution and Building the Platform

**Figure 1:**
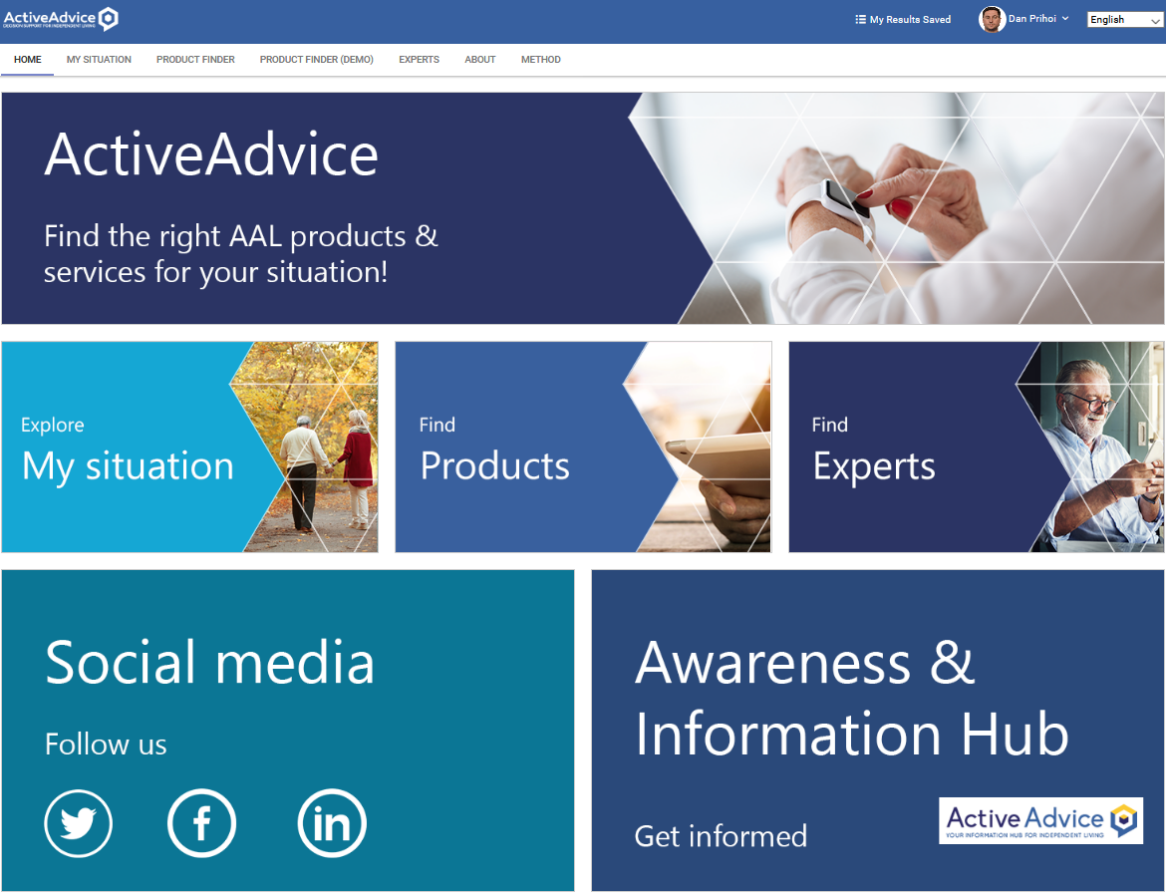
The ActiveAdvice project main index with tile-based navigation. AAL: ambient assisted living.

**Figure 2:**
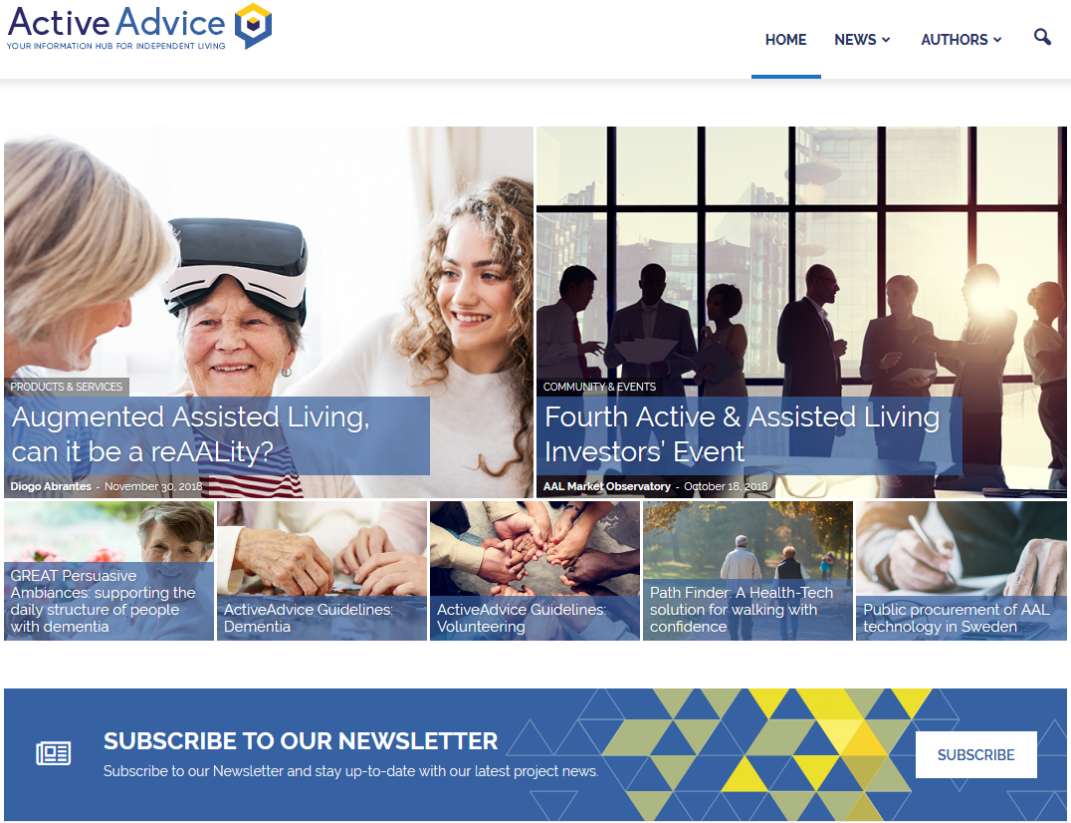
The ActiveAdvice awareness hub.

The ActiveAdvice platform was envisioned to be a product-advising, awareness hub on AAL solutions across Europe, with an emphasis on the premises of UCD and PD, drawing from the feedback of all interested parties (Figure 1). It would also feature a blog branch with assorted information on the AAL subject, including funding and support measures for the purchase of products and services, tips on community resources, articles from experts, personal stories, and other news (Figure 2).

**Figure 3:**
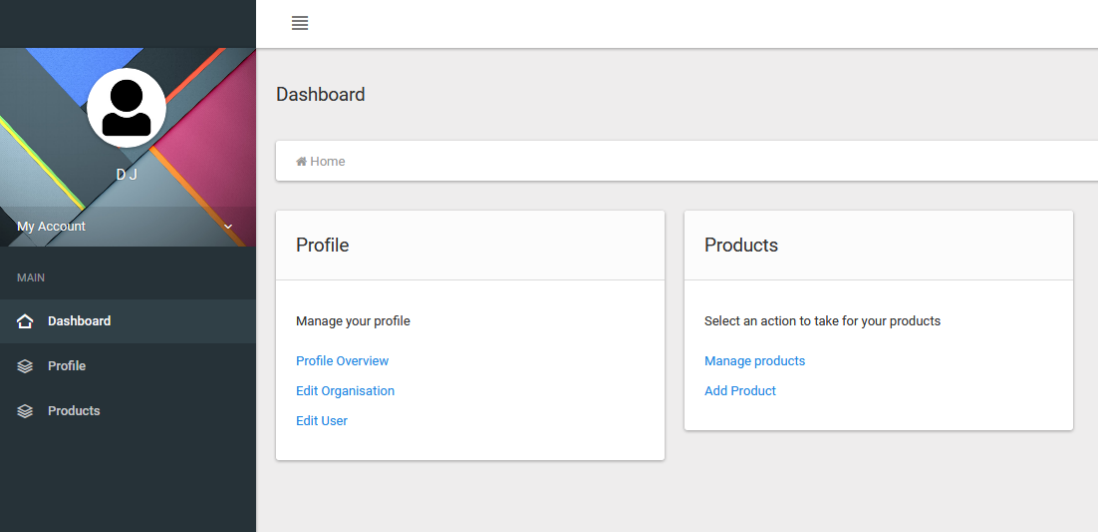
Dashboard of the business profile service module.

With functionalities being defined based on structured feedback from the requirement analysis, product cataloging was proposed following the TAALXONOMY classification system, which takes into account international definitions (eg, World Health Organization, Organisation for Economic Co-operation and Development), initiatives (eg, Building, Recruiting, And Inclusion for Diversity [BRAID], European Innovation Partnership on Active and Healthy Ageing [EIP-AHA]), and standards (eg, ISO [International Organization for Standardization] 9999) [31]. This was supported by a comprehensive information and communications technology (ICT)–based environment, presenting a broad and state-of-the art library on available AAL products and services offered at regional, national, and international levels and stored in a cloud database. For testing purposes, all available products on the platform were added by the research team. At a later stage, the catalog is populated by companies that could benefit from the dissemination of a strong and established platform by means of a service module developed for their business profiles (Figure 3).

**Figure 4:**
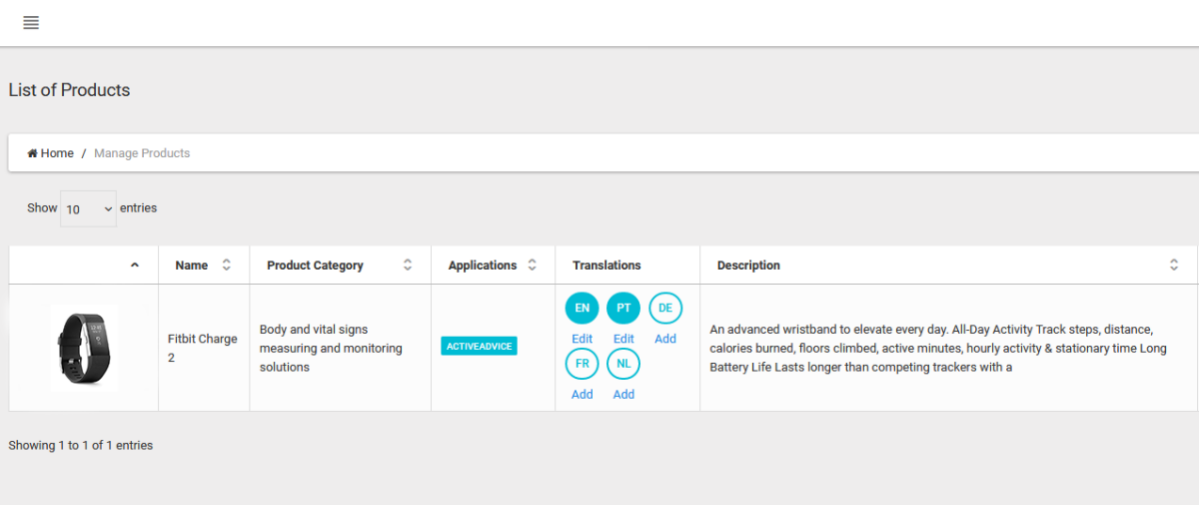
List of added products on the business module.

**Figure 5:**
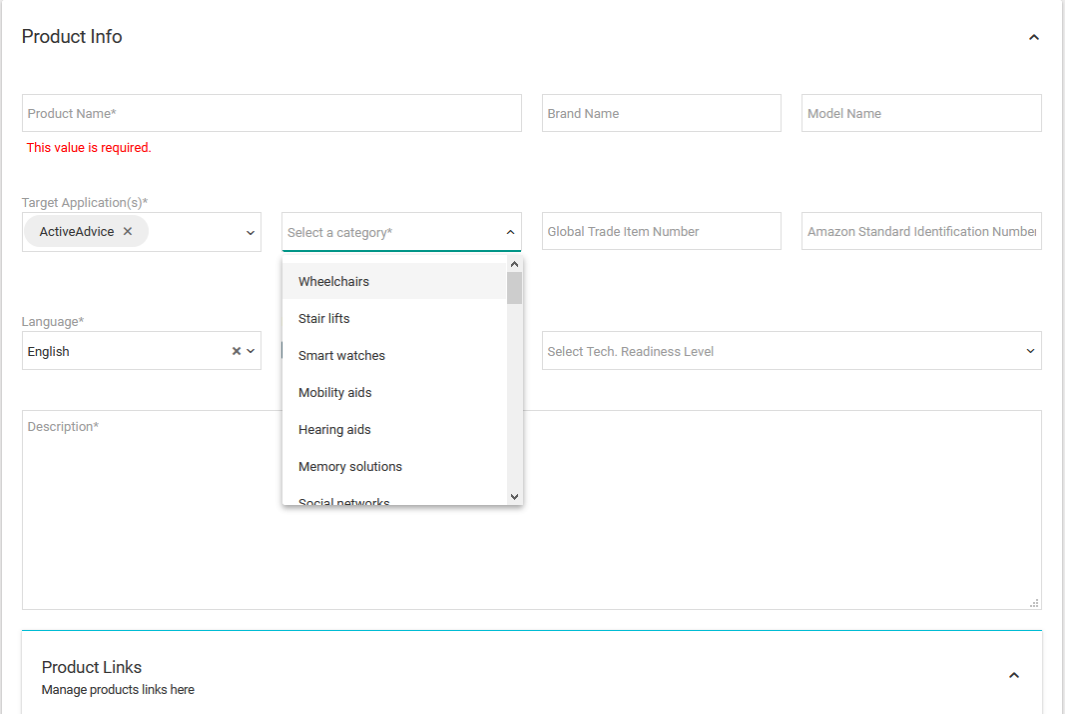
Adding a product to the database from the business module.

**Figure 6:**
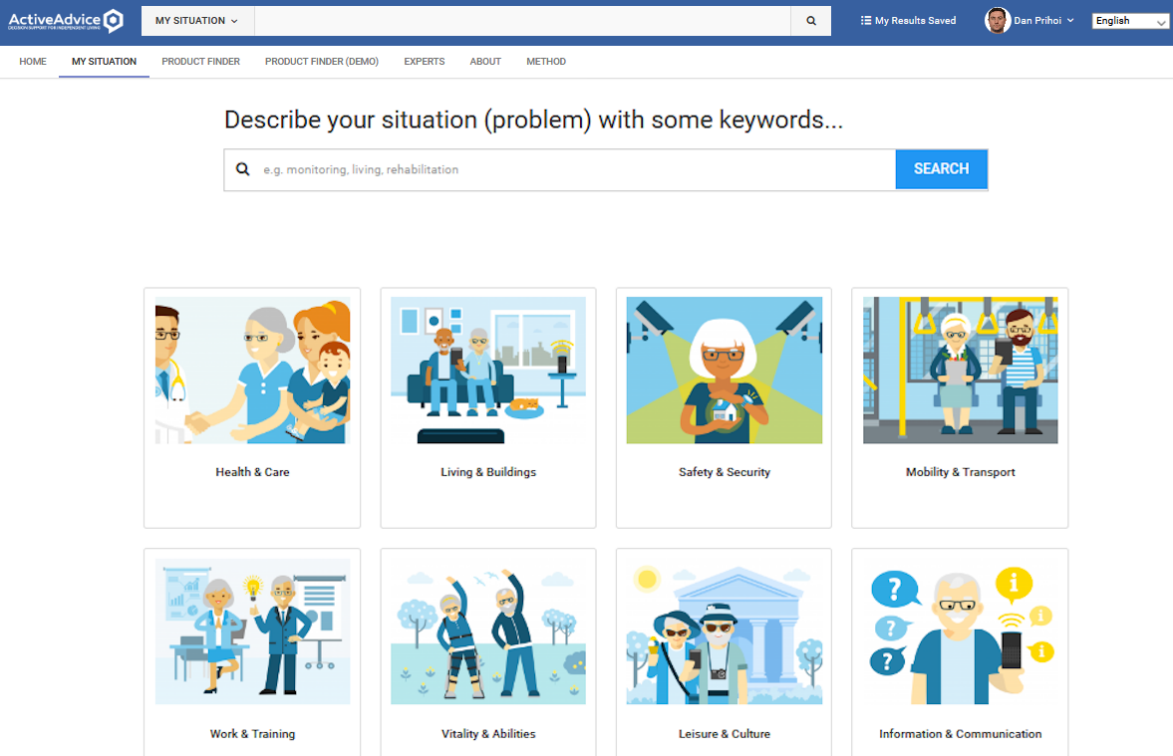
Available master categories with search bar.

**Figure 7:**
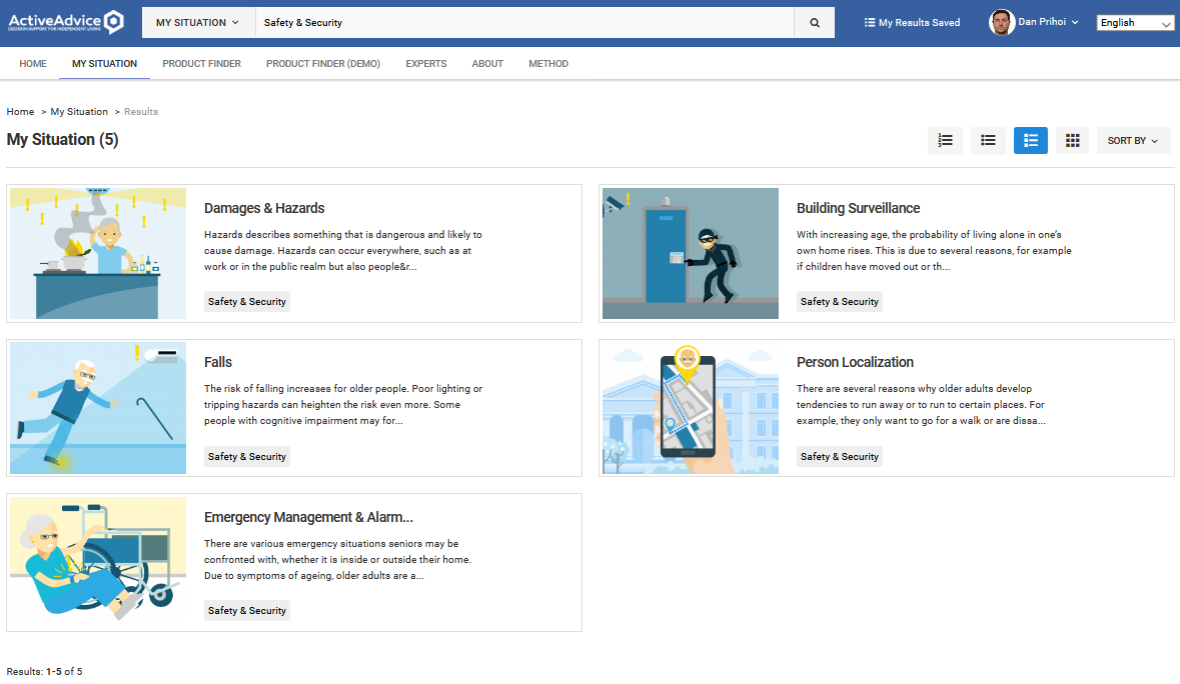
Available subcategories inside the main ones.

**Figure 8:**
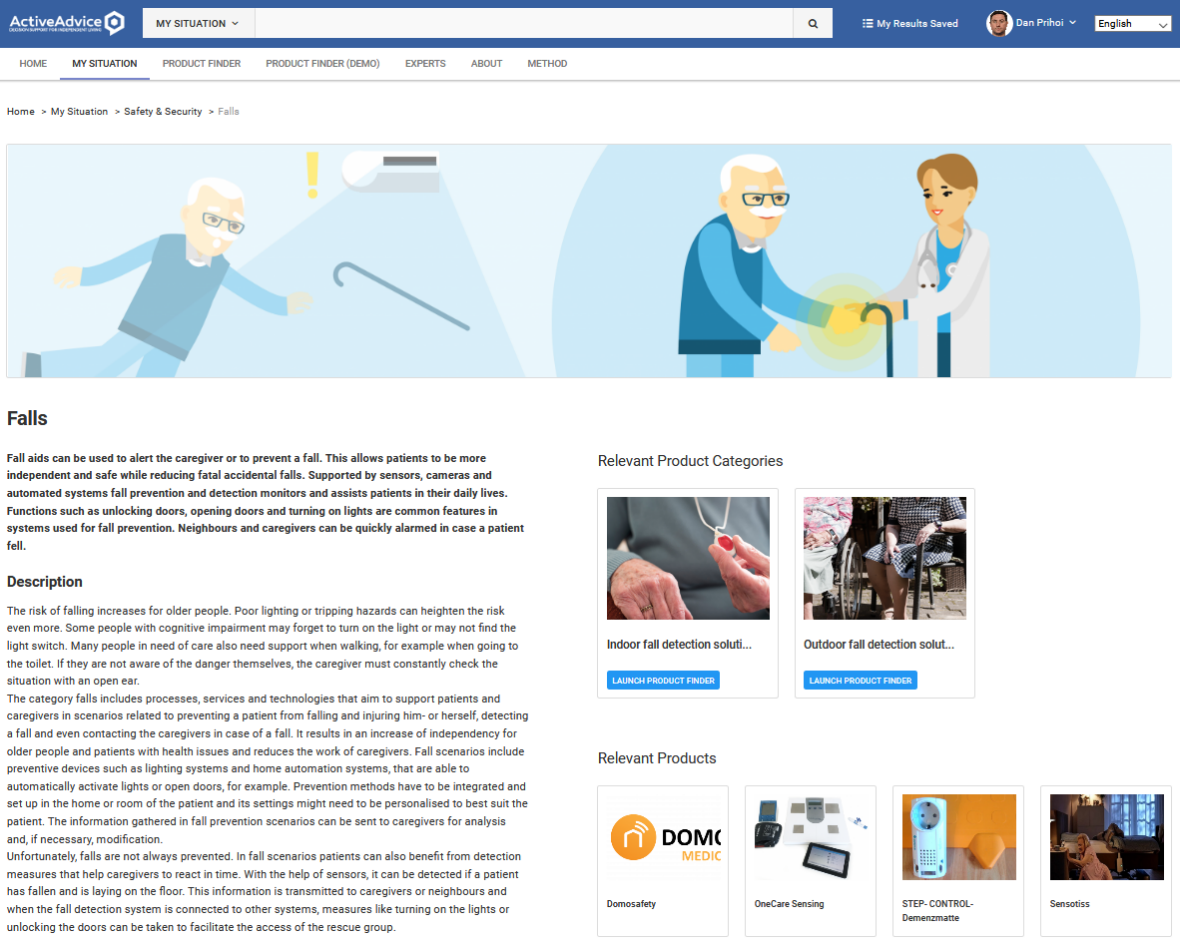
Detailed subcategory together with description and relevant products.

**Figure 9:**
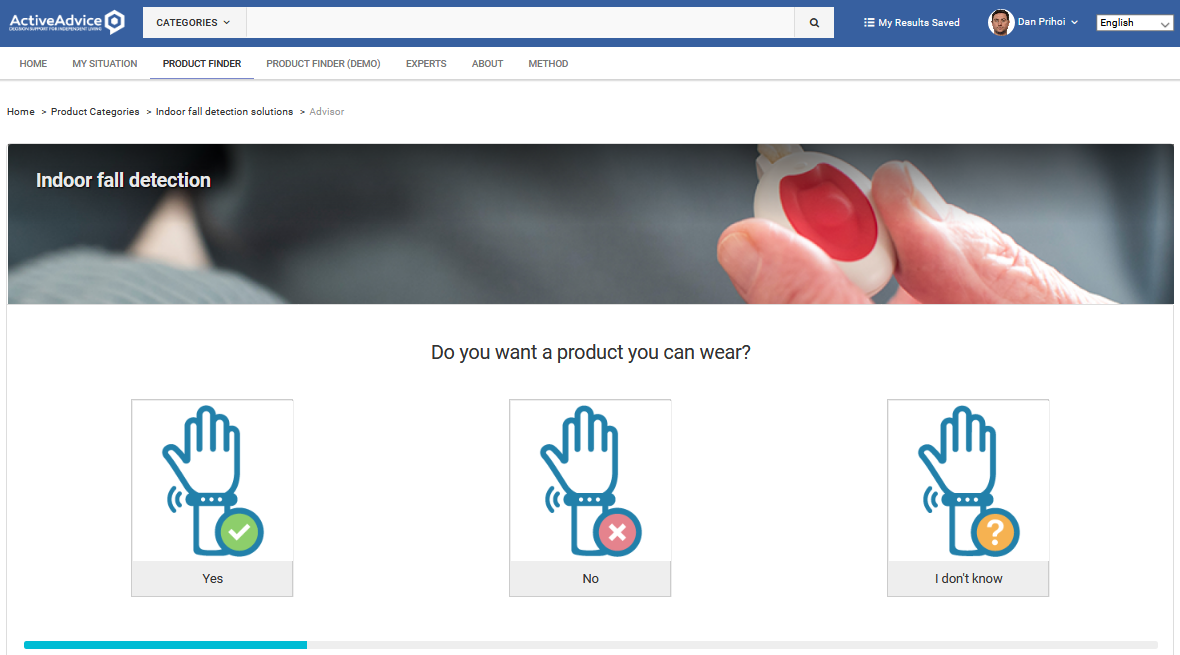
Product advisor wizard filtering product properties from user choices.

Adding a product requires filling several attributes, such as name, description, price, related features, and subfeatures (Figures 4 and 5). In this way, products are tagged with their specific characteristics, which fit them into a certain category (or categories; Figures 6-8), where they can be reached by a simple keyword search or by resorting to an advisory feature. The latter can guide the users by asking or exemplifying common or personal situations (issues, problems, conditions, and statuses) and then posing a series of questions and filters to either find a product suited to their needs and wishes or be given a suggestion for the next best option, given the availability (Figure 9).

Businesses are required to specify the type and amount of expertise they have, know-how, and technical support needed to install, upgrade, or maintain the indicated services and products, described with concise and honest data. Ideally, information should be provided not only on where to buy products or services (online or offline) but also on who can or will install them if needed. The more thorough they are in this process, the better the chance that their product will stand out among others.

With the launch of a viable prototype, the researchers set out to test how prospective end users of the ActiveAdvice solution—older adults and ICs—would interact with it. Future plans include the evaluation of not only the service module but also the general user platform interface with business profiles, along with the inclusion of government representatives, both for usability and feasibility analysis.

## Methods

### Main Approach

The development of the ActiveAdvice platform went through several internal feedback cycles and end user usability testing. The general goal for usability tests was ultimately to identify the extent to which the interface facilitated a user’s ability and motivation to navigate the platform. The usability and appeal of the design, information flow, and architecture were also analyzed based on the collection of both objective and subjective measures. These would include pre- and posttest surveys, along with a series of tasks performed by the users within the platform, while following a think-aloud protocol. Outcomes would clarify if the platform’s functionalities were aligned with the needs and expectations of end users, while presenting a high level of usability to the point where potential customers showed interest in using it. In accordance with the systematic review by Martins et al [32] on usability evaluation methods that have been used during the last few years, empirical methods are the most frequently used, which confirms the recognition of the end users’ roles as a source of knowledge for usability evaluations. In addition, a very comprehensive guideline from Nedopil et al [29] also reassured that the test procedures and methodology used in this study are in conformity with a successful process of user integration and product evaluation.

### Participants

Primary data were collected from a nonprobabilistic sample. Prospective end users of the ActiveAdvice platform were recruited by convenience and resorting to advertising or snowball sampling in Belgium, the Netherlands, Portugal, and the United Kingdom. All information collected from users was gathered exclusively for testing and improving the prototype. An informative, jargon-free sheet was provided to all participants at the time of recruitment, including a description of the study’s aims, conditions to participate, and eligibility. Participants who agreed to cooperate signed an informed consent according to the Declaration of Helsinki, with their personal data being pseudonymized, codified, and stored in secured servers to safeguard the right to privacy. Participants were free to withdraw from the project and request the deletion of their data at any time without the need for justification or incurring penalties.

Eligible individuals participating in this study were older adults living in the community (not in institutional care) and informal or nonpaid caregivers or relatives of older adults living in the community. We considered not only individuals aged 65 years or older but also young older adults (aged 55 years and older) to capture a profile of individuals who are preparing their aging process and thinking ahead. With respect to older adults, we selected individuals matching 2 distinct profiles: (1) autonomous older adults with no perceived or apparent relevant functional loss, who wish to live longer at home and think ahead to prepare for a potential loss of autonomy or upcoming chronic illnesses; and (2) older adults presently facing some degree of functional loss with implications for the autonomous performance of basic and/or instrumental activities of daily living (as self-declared), who wish to live longer at home while avoiding institutional care. Moreover, we also aimed to include informal caregivers of older adults living in the community who provide nonpaid and ongoing assistance with basic and/or instrumental activities of daily living.

Efforts were made to gather heterogeneous participants across those profiles regarding demographics (eg, age, including older and younger older adults; sex; living arrangements including older adults living in rural or urban settings) and ICT skills, in particular internet usage (including more or less ICT-savvy users). To appraise this diversity, a screening tool (described in Data Collection Tools) was used by the research team for the recruitment process.

### Data Collection Tools

The researchers conducting the usability tests were provided with a toolkit developed by the consortium, highlighting systematic instructions for recruitment, testing, and reporting as a means to guarantee consistency across countries or testers.

Regarding the user eligibility section, the toolkit included the following:

A *screening tool* to administer at the first contact with the potential participant to check for eligibility criteria and pursue a diverse sample. It included the following:

1. Questions on basic sociodemographic characteristics of potential participants (age, sex, living arrangements, years spent in education, and country of origin)
2. Questions to appraise the autonomy profile: Potential participants were asked to classify their degree of independent living and daily task accomplishment without assistance. Participants selecting “very easy” and “somewhat easy” fitted the profile of autonomous older adults. Others reporting “somewhat difficult,” “very difficult,” or “cannot accomplish daily tasks without assistance” matched the profile of older adults with autonomy loss. This question was also asked to informal caregivers about the care receiver.
3. Questions to appraise the intent or actual use of products or services to support their own everyday activities or those of the person being cared for. The participants indicated whether they need, or the person they care for, is in need of assistance from products, services, or both. In the case of a positive answer, they indicated if they were already using them, considering a purchase, actively looking, or thinking about doing so. In case there is no recognized need for products or services, participants indicated if at least they had ever thought about it preventively.
4. Questions to appraise the intent of aging in place (applied to older adults only), which included asking about the intention to continue living in their current home in a 10-year time frame, as well as about the plans to make home modifications to support aging in place.

Regarding the usability test session, the toolkit included:

1. A questionnaire featuring questions on ICT usage, including (1) a question to appraise the frequency of internet usage as daily, at least once a week, at least once a month, less than once a month, and never; (2) devices used to access internet and activities performed online; and (3) attitudes toward technology, using 9 items gathered from the Media and Technology Usage and Attitudes Scale. Only 2 attitude subscales were selected—positive and negative attitudes subscales—as the authors state that subscales can be used in isolation [33]. Items in this scale are rated from 1 (strongly disagree) to 5 (strongly agree). All questions were aimed at understanding the skills and mindsets of the participants, which could be useful in framing the findings.
2. A list of scenarios and subsequent tasks to be performed in the prototype, featuring hypothetical real-world scenarios involving needs that could be addressed by AAL solutions [34]. These scenarios and tasks were provided to participants in printed forms to facilitate immersion and provide context for the participant to engage with the interface.
3. Posttest debrief, which included open questions concerning the participant’s feedback on key features of the platform, such as the amenity of color schemes, clarity of language, the content’s level of readability, perceived usefulness, and perceived ease of use. These also included overall impressions and missing features that they would expect to see. Participants were also asked to position themselves about their overall experience with the platform on a Likert scale ranging from 1 (strongly disagree) to 7 (strongly agree), along 9 statements, using an adapted version of the System Usability Scale (SUS) [35]. The need of adapting the items on the scale created on the basis of a previous pilot experience of the usability testing protocol where the researchers concluded that some items of the original scale were not understandable by older adults (eg, “I thought there was too much inconsistency in this system,” or “I found the various functions in this system were well integrated”).

### Procedures

Tests were carried out in 4 countries: Belgium, the Netherlands, Portugal, and England. The research team in Austria was responsible for the internal testing and development. To allow testing in multiple countries and cultural contexts or groups, the platform prototype was translated into Portuguese, English, German, Dutch, and French.

Sessions were implemented in realistic but controlled environments, addressing first the core functionalities of the platform and lasting from 30 to 50 minutes. Each test session included 2 test administrators, one having the role of moderator and the other being the observer and note taker. Regarding the session schedule, the participants were first briefed about the process and asked to fill out a consent form and the ICT usage and attitudes questionnaire (section Data Collection Tools). Subsequently, they were given access to the platform and asked to engage with the proposed tasks while thinking aloud when trying to solve them.

Three different tasks were proposed, each referencing a different scenario, with the respective goals being built to understand if users could find specific products, allowing the researchers to learn how they behave. The first task asked the user to find an alarm watch—a specific product-based on the following hypothetical scenario:

Last Summer you fell down and broke your hip. You were alone, and it was very painful. Being fully recovered took you some time and you wish to prevent situations like that from happening again. You want to find a watch that works like an alarm in case of a fall. Knowing that if you fall down at home, there might be a chance that in case no one is there to help you, someone will assist you in time.

The second task asked the user to find a suitable product, using a specific functionality of the platform—a product filtering wizard-based on the hypothetical scenario:

A close friend of yours went to the doctor and was told he was starting to show some signs of Alzheimer’s, which means that he might start to become forgetful in the near future. He lives alone and still shops for groceries and likes his morning walks. Your friends’ children would feel more comfortable if he would carry some type of localization device that would allow them to know where he is, so he asked you for help to find a solution. This will help him to stay independent while giving his children some peace of mind. He would like to spend no more than 150€, and it should have an alarm function.

The third task asked the user to use the platform as they would see fit to find a generic suitable product based on the hypothetical scenario:

During a conversation with your neighbor she tells you about her heart condition, as over time it has worsened, hospitalizing her a few times. Her doctor advises her to keep track of her heart rhythm. In order to prevent risks she needs to be aware when the heart rate is spiking. The neighbor asks you to help her find a suitable product that could keep track of her heart rhythm and warns her in case it rises above a certain level. She would like to spend no more than 200€ on a solution.

Provided with these contexts, the participants would look for a way to obtain the aforementioned product while navigating the platform. The process itself, along with their verbalizations, would provide the researchers with an idea of the difficulty level as well as other inherent parameters regarding layout and organization. The level of success regarding task completion (the moment when the user finds the targeted product) was registered by the researchers according to a 3-point classification: 0=not completed; 1=completed with difficulty or acceptable prompts; and 2=easily completed. The *stop* criterion for task performance was applied when 1 of 3 conditions occurred: (1) the users completed the task successfully; (2) the users said they completed the task, even if they did not; or (3) the users decided to give up. During task performance, the users’ navigational choices, verbalizations, and nonverbal reactions (eg, facial expressions) were registered and recorded *verbatim* by resorting to written notes, video screen capture, or audio capture.

All researchers were provided with a list of all possible pathways to successfully complete each task to facilitate the recording. Finally, after task completion, the posttest debrief (*Data Collection Tools*) was administered to gather participants’ perceptions of the platform and satisfaction. The audio recordings of the posttest interviews were transcribed by researchers from each country performing the tests and collecting the data.

### Data Analysis

Descriptive statistics were used to characterize the participants in this study as well as to describe quantitative usability indicators (eg, ratings on scales). Absolute and relative frequencies, central tendency, and dispersion measures were used as appropriate. When reporting median values, the IQR was the measure of statistical dispersion selected to be reported. The administered scales were analyzed itemwise. In particular, for the adapted version of the SUS, a composite score for the 9 items was not computed, as we could not assume unmeasured psychometric properties of the scale (eg, factorial structure).

After transcription, text data, both from relevant user verbalizations when performing the tasks (think-aloud protocol) and from the interviews on the posttest debrief were analyzed by performing a thematic analysis [36]. The themes were defined deductively, meaning that they were guided by a structured analysis matrix [37]. The matrix corresponded with the main topics approached in the posttest interview guide (refer to *Data Collection Tools*). The deductive or top-down approach in data analysis was selected as the most suitable and feasible approach to guarantee a common ground among researchers from different nationalities. A researcher from each country performing the tests performed this analysis; thus, 4 researchers performed the tests. Next, the contents categorized in each theme were translated from the source language to English by researchers from each country. All excerpts organized in their respective categories were sent to the Portuguese project team, who created a common data file. The entire corpus was inspected by a researcher of this team to find any inconsistencies among categories and correct them if necessary, under the agreement of the researchers who first analyzed the data. This is a validation process for a deductive approach to data analysis [37]. A researcher from the Portuguese team then identified, if any, the trends, or most common opinions within each theme, inspected for potential different trends per group (eg, trends per country, per type of participants—older adult or IC) and reported the overall findings. In the *Results* section of this paper, relevant text excerpts are used to illustrate themes and trends.

## Results

### Sociodemographic Characteristics of the Participants

A total of 32 participants matching any of the described profiles were recruited. Of these 32 participants, 21 were older adults, and 11 were informal caregivers or relatives of older adults. The former group was composed of 13 female and 8 male participants, and the latter by 4 female and 7 male participants. Overall, the sample was well balanced with regard to sex (17 female and 15 male), although within the groups of older adults and caregivers, the sex distribution was less balanced with more female than male participants in the former group and the opposite trend in the latter. Regarding age groups, participants were distributed from a 25-29 years range to an 80 years or older range; most older adults were aged between 65 and 79 years (12/21, 57%) and most caregivers were aged between 55 and 64 years (6/11, 55%). The participants’ education level was rather high, with a median of 15 years of formal education, either for the group of older adults (IQR 6.5) and for informal caregivers (IQR 4; median of 15 years for the entire sample, IQR 5.75). Most users (20/32, 63%), regardless of being older adults or caregivers, lived in a household of 2; 12 (12/21, 57%) of them were older adults, whereas 8 (8/11, 73%) were caregivers. Regarding the 2 profiles of older adults we aimed to recruit (see *Participants* section), most of them were autonomous, who wished to prepare for a future functional decline. When older adults (n=21) assessed the extent to which it was easy or difficult for them to live independently and accomplish daily tasks without assistance, most classified it as “very easy” (14/21, 67%), 3 as “somewhat easy” (3/21, 14%), 2 as “somewhat difficult” (2/21, 10%), 1 as “very difficult” (1/21, 5%) with 1 older adult reporting to “not being able to accomplish daily tasks without assistance” (1/21, 5%). Caregiver-wise (n=11), 3 reported that the older person they support “cannot accomplish daily activities without assistance” (3/11, 27%), the same number reported a *very difficult* accomplishment of such activities (3/11, 27%), and 2 caregivers reported “some difficulty” (2/11, 18%). In contrast, 2 caregivers (2/11, 18%) classified as “very easy” and 1 as “easy” (1/11, 9%) for the autonomous completion of daily activities by the person they support. The country mostly contributing to the recruitment of older adults was the Netherlands (13/21, 62%), as it is a country with good digital literacy among older adults [38]. Portugal contributed the most to the recruitment of informal caregivers (6/11, 55%), as it is a country characterized by a familialistic approach to care provision (the so-called Mediterranean care model [39,40]). Further details are presented in Table 1.

**Table 1:** Demographic data from the study participants (N=32).

Variable			Older adults (n=21)		Informal caregivers (n=11)
**Age group, n (%)**					
	<55	0 (0)		1 (9)	
	55-64	8 (38)		6 (55)	
	65-79	12 (57)		1 (9)	
	≥80	1 (5)		3 (27)	
**Sex, n (%)**					
	Male	8 (38)		7 (64)	
	Female	13 (62)		4 (36)	
Years spent in education, median (IQR)			15 (7)		15 (4)
**Autonomy profile, n (%)**					
	Autonomous or no relevant functional decline	17 (81)		8 (73)^a^	
	Loss of autonomy or relevant functional decline	4 (19)		3 (27)^a^	
**Living arrangements, n (%)**					
	Living alone	7 (33)		1 (9)	
	Household of 2	12 (57)		8 (73)	
	Household of 3 or more	2 (10)		2 (18)	
**Country of origin, n (%)**					
	Belgium	5 (24)		2 (18)	
	The Netherlands	13 (62)		2 (18)	
	Portugal	2 (10)		6 (55)	
	United Kingdom	1 (5)		1 (9)	

^a^The autonomy profile in the column of informal caregivers refers to how they appraised the person they care for, thus referring to the autonomy profile of the person they care for.

### Plans to Age in Place

Regarding plans to age in place, the great majority of older adults in this study (17/21, 81%) declared their intention to continue living in their current home in the next decade, with 14 (14/21, 82%) of them contemplating the modification of their current houses to achieve that. Participants planning to move (4/21, 19%) were all autonomous older adults at the time of data collection. More than half of all participants (56%, n=18) reported a need to use products, services, or both to support the performance of their own daily activities or the activities of the person they care for. Curiously, among those, one-third (n=6) reported an “easy” or “very easy” completion of daily activities (their own or of the person receiving support) without help, suggesting a *think-ahead* mindset. Among those recognizing the need for supportive products and/or services (n=18), 8 (8/18, 44%) reported that they had already used these types of solutions, 11% (2/18) already found what they needed and were considering a purchase; 22% (4/18) were actively looking for the most suitable solution, and 22% (4/18) had yet to take any action to look for solutions. Among the participants currently using, having found, or actively looking for supportive services (n=14), only 4 (4/14, 29%) had resorted to ICT-supported products and/or services. From the participants who did not report a need of using products and/or services to support their own or their care receivers’ daily activities (14/32, 44%), more than half (8/14, 57%) showed a preventive mindset, having thought about using such products or services to facilitate aging in place.

### ICT Usage and Attitudes Toward Technology

The majority of participants (29/32, 91%) stated that they used the internet on a daily basis, with the remaining participants (3/32, 9%) using it at least once a week. The tablet was the most used device to access the internet (24/32, 75%), followed by the smartphone (20/32, 63%) and the laptop (18/32, 56%), with the desktop being the lesser used device (15/32, 47%). As for online activities, consulting news and weather reports (30/32, 94%), email and online messaging (29/32, 91%), and looking for products and services information (24/32, 75%) comprised the top 3, whereas online banking (20/32, 63%) and social media (20/32, 63%) fell on the sparser habits.

Concerning attitudes toward technology (Table 2), there were some paradoxical findings with users showing both overall positive (items 1, 2, 3, 4, and 5; median rates from 4 to 5) and negative attitudes toward technology (items 7, 8, and 9; median rates of 4). A substantial share of the users (28/32, 88%) agreed with the importance of having access to the internet at any time and to information offered online (median of 5). More than half of the participants believed that technology offers a solution to many problems (26/32, 81%), stated that they like to keep up with technological trends (23/32, 72%), and feel more accomplished with the use of technology (20/32, 63%; all median of 4). Less than half of the participants believed that technology complicates life (15/32, 47%), but a relevant share believed that technology makes people waste too much time (20/32, 63%) or that it might contribute to increased personal isolation (20/32, 62%, all median 4).

**Table 2:** Participant’s attitudes toward technology (N=32).^a^

Item	Participants, n (%)				Participants, median
	(Strongly) disagree	Neutral	(Strongly) agree		
1. Important to find information online	0 (0)	4 (12)	28 (88)	5^b^	
2. Important to access internet at any time	4 (12)	0 (0)	28 (88)	5^b^	
3. Important to keep up with technological trends	4 (12)	5 (16)	23 (72)	4^b^	
4. Technology is a solution to many problems	2 (6)	4 (12)	26 (81)	4^b^	
5. Technology makes anything possible	3 (10)	20 (63)	9 (29)	3^b^	
6. Technology helps to feel accomplished	6 (19)	6 (19)	20 (63)	4^b^	
7. Technology makes people waste time	6 (19)	6 (19)	20 (63)	4^c^	
8. Technology complicates life	6 (19)	11 (34)	15 (47)	4^c^	
9. Technology increases isolation	5 (15)	7 (22)	20 (63)	4^c^	

^a^The description of the items is abbreviated and rephrased for presentation purposes. To learn about the scale from where these items were gathered (Media and Technology Usage and Attitudes Scale, positive and negative attitudes subscales, please consult Rosen et al, 2015 [33]).

^b^Scale ranges from 1 to 5, with higher median values indicating more positive attitudes.

^c^Scale ranges from 1 to 5, with higher median values indicating more negative attitudes.

### Usability and User Impressions

#### Task Analysis

Regarding successful tasks with no input from the researchers (Table 3), task 3 had the best results (15/32, 47% success rate), followed closely by task 1 (14/32, 44% success rate). However, if we refer to the completion of the task at hand, be it by the user alone or with acceptable prompts from the researcher, the most successful was task 2 (26/32, 81% full and partial success rates). Accordingly, task 3 also had a higher rate of unsuccessful completion (10/32, 31% no success rate), followed by task 1 (8/32, 25% no success rate).

**Table 3:** Task completion rates and illustrative verbalizations on task (N=32).

Task	Participants, n (%)				User verbalizations
	Full success	Partial success	No success		
Task 1: use the platform to find an alarm watch	14 (44)	10 (31)	8 (25)	“I'm not sure that it is what I'm looking for, I’m not sure of being in the right section”; “There is a lot of overlap between the various categories”; “I would search for the name of the device on the bar, but I don’t know the term, so I’m trying to find a relatable category”	
Task 2: use the product finder to find a device that allows you to locate a person	12 (38)	14 (44)	6 (19)	“It is not clear for me how to deal with the filtering questions and the price drag bar”; “The problem should be discussed first, and then it would present you with choices”	
Task 3: use the platform to find a product that suits the need of your neighbor	15 (47)	7 (22)	10 (31)	“This demands a lot of knowledge on these products to find the desired product”; “It is hard to come up with keywords”; “I don’t really think that some people would know what some of these options/functions actually mean”	

#### Posttest Debrief

Overall, participants’ ratings of their experience with the ActiveAdvice prototype with regard to usability were fairly positive (Table 4). The usefulness of the platform was by far its best appraised characteristic, with the great majority of participants considering the ActiveAdvice platform to be a useful resource (29/32, 91%). More than half of the participants considered that the platform was easy to use (19/32, 59%) and well organized (21/32, 66%) and that information on the platform could be easily retrieved (19/32, 59%) and navigating on the platform was a pleasurable experience (21/32, 66%). Most participants also considered that navigating on the platform could be done with no support from others (20/32, 62%) and that it was easy to keep track of their location within the platform (19/32, 59%). However, participants assumed an overall neutral position when judging how easy it would be for other people to use the platform, with less than half agreeing that most people would quickly learn how to use the website (15/32, 47%). Despite the overall good appraisal of ActiveAdvice usability, less than half of the participants reported that they would like to use the platform frequently (13/32, 41%).

**Table 4:** Ratings of the users’ experiences with the ActiveAdvice interface (N=32).

Item	Participants, n (%)				Participants, median
	(Strongly) disagree	Neutral	(Strongly) agree		
1. I think the website was easy to use.	4 (13)	9 (28)	19 (59)	4^a^	
2. I think I would need support to be able to use this website.	20 (62)	3 (9)	9 (28)	2^b^	
3. I think the website is well organized.	8 (25)	3 (9)	21 (66)	4^a^	
4. I could get the information quickly.	8 (25)	5 (16)	19 (59)	4^a^	
5. I found it difficult to keep track of where I was on the website.	19 (59)	4 (13)	9 (28)	2^b^	
6. I think that most people would learn to use this system very quickly.	11 (34)	6 (19)	15 (47)	3^a^	
7. I think that I would like to use this website frequently.	9 (28)	10 (31)	13 (41)	3^a^	
8. I think the website is useful.	3 (9)	0 (0)	29 (91)	4^a^	
9. I found the website pleasant to use.	7 (22)	4 (13)	21 (66)	4^a^	

^a^Scale ranges from 1 to 5, with higher median values indicating a more positive assessment of usability.

^b^Scale ranges from 1 to 5, with higher median values indicating a more negative assessment of usability.

The quantitative assessment of users’ experiences (ratings) was further explored by collecting qualitative data from a posttest debrief or interview. We provide a qualitative synthesis of the content produced by the participants with respect to each specific dimension of the platform. The debriefing was conducted in a semistructured style, thus not all participants contributed to each unique topic under the analysis below. We did not distinguish between quotes from older adults or caregivers, as no different patterns emerged from their answers to the posttest interview.

#### Layout (General Impressions)

Overall, the ActiveAdvice layout was well accepted by most participants (17 of 23) and considered to have an organized and clear structure:

Clear structure and well organized.

User (U)7

I think it’s pretty and well organized.

U18

It’s very clear and neat.

U10

A total of 6 participants perceived negative aspects of the layout, considering the platform complex or confusing, while also including too many product categories:

Could be better, I had to think where I was a lot of times.

U17

It’s neat and low-key. There are a lot of categories though.

U11

Stick to simple things.

U16

#### Amenity of Color Schemes and Use of Images

Most participants were pleased with the esthetics of the platform (23 of 27):

Not flashy, creates serenity.

U9

The use of colour is soothing.

U14

I found the colours very appealing.

U20

Four participants found the platform “a bit too bland” [U3] or “not contrasting enough” [U4]. Images were considered attractive, conveying a nondiscriminatory image of older adults or disabled or ill people:

the platform is developed not only for elderly people and it does not feed stereotypes about elderly people, this could be for anyone, a family with elderly or someone with some disability who wants to live independently (...) the appearance makes me think that.

U24

[images are] not offensive.

U22

#### Readability

This parameter allows participants to appraise how easy it is for them to read and understand the text on the platform, depending both on the presentation of text (eg, font size, spacing) and the actual text content (ie, use of understandable words and sentences). Regarding text presentation, participants were divided into those who appraised this parameter positively (14 of 23):

...easily readable.

U3

...style is professional and easy to read.

U7

Some participants who did not appraised this parameter positively (9 of 23). Participants with negative comments mostly focused on font size, and 1 participant mentioned the amount of text:

...text size in some areas is too small.

U5

...too much text.

U21

Regarding textual content, most participants considered them easily understandable (19 of 25):

...good, sentences were written in short and plain language.

U22

...terminology is fine.

U14

...very perceivable.

U7

Negative comments (6 of 25) relied on difficulties in understanding some of the terms used to describe the solutions:

...some words are difficult to understand.

U15

...for people with lower education one should be careful in using no professional terms.

U23

#### Missing Features

When requested to point out any missing functionality or feature in the ActiveAdvice platform that would be expected, users were divided into those who reported not missing anything in particular (12 of 25) and those stressing a number of different expectations, which include more feedback, more support, and more product information:

I would like to get more feedback on my actions and be able to really access the products.

U12

...product information should be much better.

U9

...there should be a number you can call if you need help figuring out how the website works.

...people who use the website would be able to ask questions or exchange information, that would be very interesting.

U19

#### Most and Least Liked Features

When questioned about the pluses and the most liked features of the platform, participants stressed the usefulness and purpose of the platform (13 of 27) and its ease of use (8 of 27), nice layout, and overall appearance (11 of 27):

Good for older people who start to have trouble living at home.

U5

The concept is very interesting and could be useful.

U10

It’s faster than go to a physical store and compare.

U12

I think the overall impression is that it looks good. I like the pictures.

U13

It’s a site of easy access and lets people know about what is available.

U19

Intuitive, if you get to know it a little bit then suddenly it becomes very easy to use.

U23

In contrast, platform minuses almost exclusively focused on missing a more effective product search and more complete product information (10 of 27):

The filtering comes too late in the process of searching. Should be earlier in the process.

U6

The filter questions I think. You should be able to filter on your problem.

U8

More information about categories without having to click on them.

U11

Lacks keywords so you can use the search function. Can’t see the difference between scenarios and categories.

U22

## Discussion

### Principal Findings

Our study presents an innovative platform in the AAL field, ActiveAdvice, and provides observations on the usability and users’ experiences within the prototype. Studies on innovative platforms, such as ActiveAdvice, are often missing in the literature; thus, this work contributes not only to improving the prototype but also to populating the literature in this field. The main insights from this study are derived from both its results and the process, which are discussed below in a series of comments and takeaways over 2 critical sections of this work: the usability tests and the process as a whole, which translates our thoughts from the gathered data. The first section rests on dissecting the participants’ attitudes and achievements while using the platform and how those observations lead to changes in the interface or information architecture. These insights are the core of knowing how to build something for a specific audience with different needs and ways to interpret digital media. Impressions on the experiences are also of crucial importance, as the reception, interpretation, usefulness, and perceived user satisfaction and engagement are effectively and ultimately the make-it-or-break-it of what we set out to develop. The second section discusses every limitation and interesting findings regarding the entire process and the characteristics of the recruited users, along with possible explanations for the obtained outcomes. Both sections also aim to discern important aspects such as difficulties, remarks, and other perceptions that are structured to help others not only to replicate the process but also to take special precautions and careful considerations regarding the specified elements.

### Usability Testing

The usability testing protocol established for this study included a task analysis methodology as a means to understand users by observing them interacting with the platform while trying to achieve an intended goal. The three main tasks assigned to the participants revolved around searching products while using the existing functionalities of the ActiveAdvice platform, as these are considered the core tasks that the website must support.

We saw that task success *with ease* and *with help* were sometimes on par with each other, meaning that although the functionality was there, a fair number of users would struggle to complete the task because of confusion or lack of a clear direction. Each scenario was described in a way that allowed a very spontaneous search to look for natural patterns of interaction or provided the user with more structure to incentivize the use of specific components (eg, asked to use a certain search functionality) or named the solution being searched (eg, task 1, a smartwatch). When less detailed scenarios were provided (eg, task 3), the chances of failure were higher. Users showed a substantial lack of knowledge on AAL products or services, thereby increasing the difficulty in thinking about keywords or categories to which they could resort to for finding a solution to their needs. This observation is well illustrated by the participants’ verbalizations in Table 3. This finding is particularly relevant to the AAL field, provided the considerable lack of public awareness of such solutions, and the complexity characterizing most AAL solutions (commonly requiring integration of products and services, frequently from different providers). Thus, the challenges faced in building a platform such as ActiveAdvice, where the user should be able to find a suitable solution to their problem, are more pronounced than in similar service platforms for other markets. The resource to typical search and filtering methods (eg, keywords, categories, list of product or service features) is not sufficient in terms of efficacy, as users frequently do not possess a sufficient understanding of ICT or ICT-enabled solutions to choose keywords, decide on search categories, or whether a technical feature on a device is or is not relevant for them. This observation has implications for not only the design of the ActiveAdvice platform but also initiatives aimed at informing older adults on AAL solutions and promoting their uptake. From these observations, we not only introduced improvements in the interface but also identified a need to integrate digital and human advisory services to tackle complexity and improve the user’s experience (see the concept of the Authorized Active Advisor [41]). Process-wise, one should stress that when evaluating tasks, failures and successes could be somewhat misleading, as task success or failure will rest on the evaluator’s interpretation of the user’s actions. We aimed to minimize this by providing structured guidance to test carriers, but variability in these judgments cannot be ruled out. Some evaluators are more lenient and others stricter, as a *barely* could be a *fail* and an *easy* by other standards could be a *difficult*. In addition, when evaluating task completion rates, one should also not completely rely on success percentage fluctuations, as these might also be explained by concatenated success (or failure), where a user who eventually spends more time on the previous task learns how to perform better on the next one. It is not easy to avoid this type of bias because tasks are ultimately not independent. Nevertheless, the overall high task completion rates suggested that tasks were permissive in terms of global interaction.

Subjective user impressions of the prototype have been favorable overall with regard to its usability. The most encouraging finding from both ratings and verbalizations was the recognition of the usefulness of a platform such as ActiveAdvice, proving its concept. Design-wise, the platform was well assessed with regard to its layout and pleasurable navigation. However, when considering room for improvement, we should not neglect that about one-third of the users have found problems in the organization of the platform, on finding the information quickly, and on keeping track of their position on the website. Moreover, about one-third believed that support would be required to use the platform and that most people would need some time to learn how to use the system. Overall, with regard to the main quality components of a system usability, most encouraging results were found on user satisfaction; ultimately, participants had a satisfying experience when navigating the platform, mostly brought by the recognition of a *sense of purpose*. The platform was rated as appealing to the eye, whereas the scope was reported as interesting, educative, and useful, with a broad range of content. In contrast, the tests advocated a need for improvements, mainly in terms of learnability and effectiveness. Issues with general visualization or organization can contribute significantly to this subject if the interface fails to conduct the intended interaction. This is hinged on two aspects: a strong information architecture, which is tricky when talking about a higher range of product categorization and self-explanatory elements. Some products might be difficult to place, even when using a tested taxonomy, as they might span more than 1 category. That being said, the integration of a wide range of recognizable keywords takes a big share of the process when it comes to finding them quickly. Keywords that spring to mind should have immediate (and accurate) correspondence in a search bar, as the user's mental model is a crucial part of a search method. In this case, knowing and learning the most common issues the users are experiencing or will experience helps when associating those loose keywords with frequently asked questions (or frequently posed statements) in the process of finding a solution. In addition, a clear and usable design can be achieved through familiarity, consistency, guidance, and direct feedback. The participants preferred a center-focused interface, tile-based navigation, hinting at an *over-the-belt* experience, while objecting to the need to scroll down to access content. A lack of previous domain or product information knowledge makes it difficult for the user to even know what to look for or search, so a short presentation statement along with a direct visualization of what is important would help to minimize those feelings. Taking, for example, the product advisor, using a wizard, was especially handy as it simplified the task into a sequence of chunks, while dropping the learning curve, making the users follow a step-by-step path to accomplish their goal. Using polished visual cues, such as coherent images with associated labels and recognizable icons with expected placements helped to simplify the navigation. Although the use of plain terms was praised by users and accomplished in most navigational settings, it can be difficult to manage in some sections that will have to get technical to deliver the correct information across. In that case, it would be better to be explicit and true to the description than risking an oversimplified explanation that will not be up to par.

Despite a good overall satisfaction with the platform, only 42% (13/32) reported that they would use the platform frequently. However, this result may be related to sample features: first, there was a percentage of users who reported not having a real need to use products and/or services to support their own or their care receivers’ daily activities (14/32, 44%), which meant that the benefits of AAL were not sufficiently exposed to the point that peaks general interest in discovering them. Moreover, most of the older adults’ sample was composed of individuals with no functional loss and autonomous in their daily activities, meaning that they might recognize, as a double standard, the ActiveAdvice platform as useful for older adults but not for themselves. In contrast, we also have to link the fact that the platform was at an early stage of development, meaning that its interface and functionalities were not optimized at the time of testing. It was expected that failures within tasks and other inabilities to interact with the platform would cause some disappointments in some users, influencing their intention to return. The purpose of testing is to evaluate user interaction, so it is also expected that all aspects would be corrected and refined according to the participant's usage, to the point where user-friendliness would subsequently become a positive decisive factor. A pleasant experience could be what, together with an actual need, might keep users interested and susceptible to make some effort (both time and persistence wise) while using technology that they might not be at ease with, hoping that ultimately it will help increase their quality of life.

### About the Process

The interesting findings of this study must be considered within the context of some limitations. One of them concerns the selection and inclusion of only primary and secondary end users when testing the platform. Interested parties in the AAL field have been mainly identified as 3 diverse groups [42-44], with the first and biggest target of technologies and services being the consumers. The other 2 groups are governments, such as city administrations who define policies and provide services in the field of health and care, and the businesses that develop, exploit, or market products and services. In previous stages of the ActiveAdvice research and platform development (eg, requirement analysis; see the *ActiveAdvice Platform* section), representatives of all these groups were included, and the authors have argued for this multiperspective approach on AAL research [30]. For this usability study, however, although older adults and caregivers’ requirements previously gathered were mostly addressed and implemented in the tested prototype, requirements from business and government representatives (eg, specific modules and functionalities) were still in progress. Future research on the ActiveAdvice platform must enroll multiple interested parties in such a solution. Indeed, a platform that benefits more than one party needs to take into account that, apart from the common interest in the area, each group has different ways of expressing wants and needs. For example, from a budgetary perspective, governments are concerned about ensuring the successful adoption of these technologies, as full-time home or institutional health care could become very expensive. Accordingly, the promulgation and growth of the area provides businesses with a better sense of the market’s wants and needs, thus boosting research and manufacturing of products, as well as supporting a broader supply of services [30,42,43]. Another limitation was the education level of the participants, which was high (median of 15 years of schooling), causing the sample to lack diversity in this aspect. Older adults and informal caregivers were comparable in terms of education, which might have influenced the analysis, but the researchers qualitatively looked for different patterns of responses between both groups and found none. Education is a well-known determinant of internet usage, so an educated sample was expected, and the so-called education bias is common in internet- and technology-related research [44]. Our participants were frequent internet users, comfortable with a range of devices, and performed multiple online activities. They have also shown overall positive attitudes toward technology and were very open to use or already using products and/or services to support aging in place, although only a small percentage use or plan to use ICT-enabled products. Altogether, this suggests that we recruited mostly a profile of early adopters, who are not only more ICT savvy but also are more open to adopting new ideas and use innovations than their counterparts. If we consider that most participants in this sample were aged 60 years and older and attend to known statistics on ICT use by older people, this idea is further reinforced. In 2016, only 45% of users aged 65 to 74 years used the internet at least once a week, compared with 82% in the general adult population [45]. According to a survey performed in the United States [46], 34% of older internet users reported that they had little to no confidence in their ability to use electronic devices, whereas 73% revealed that they will likely need help from someone else to use an electronic device. Future work with the ActiveAdvice platform must consider the inclusion of a more diverse sample. This also applies to the autonomy status of older adults, as it was very challenging to recruit dependent older adults, and those were mostly represented in this sample by informal caregivers. In practical terms, however, the use of a platform such as ActiveAdvice to support dependent older adults will most likely be enabled by informal caregivers, especially when autonomy loss is propelled by cognitive impairments. In this study, we decided to include those designated by some authors as young older adults (>55 years) [47]. Although this conceptualization of older adults is not consensual, when considering testing of technological solutions that will only be in the market in a few years, including prospective users is important. Moreover, AAL technologies are not only intended to compensate for functional loss, most common in old age but also to prevent it; thus, including a group who thinks ahead about aging in place is fundamental to assess the ActiveAdvice platform.

Regarding the testing protocol, challenges emerged when performing tests in 4 different countries. Even with the provision of a package with a standardized toolset, one should definitely not assume that everything will go according to plan and that testers will have the same understanding of procedures, as discussed above with regard to judging a task failure or success. Moreover, owing to language barriers, we were not able to have the same researchers performing the analysis of the text data in the original transcriptions, which were first translated into English. It might be that some discourse details may be lost in translation. These are well-known challenges of European projects, and our experience shows that communication and a systematic registration of collected data in its *as raw as possible* state is key. In this study, we were not able to perform any comparison among countries: small numbers hinder hypothesis testing, and for qualitative analysis, no different pattern of responses per country emerged. Studies have reported cultural differences in how usability is assessed by users, suggesting that this is a multidimensional concept [48]. Future ActiveAdvice studies must look to the extent to which different attributes are equally valued across cultures.

Our experience has also shown that dealing with older adults when performing usability tests also has its peculiarities, as agreed with in other studies [49-51]: users may be uncomfortable discussing their personal circumstances and often reluctant to acknowledge what they consider their own frailties or shortcomings. This can pair up with being afraid of giving a wrong answer, which eventually translates into being less prone to explore the application and task at hand. In addition, as they usually want to please the evaluator and not be a burden (social desirability bias), they are less likely to make a negative remark about something even if they are struggling, resulting in short and uninformative responses that should be taken with a grain of salt. This can be improved by starting to involve users early in a PD process, where they can feel safe when performing tasks or sharing opinions.

### Conclusions

ActiveAdvice is a type of platform in the European context providing a holistic market overview and advice on AAL products and services, and its development process was tied to good practices in UCD. This platform has the potential to support the problematic uptake of digital solutions by older adults, which may support their aging in place. ActiveAdvice’s purpose is fully aligned with wider political and strategic agendas and priorities for healthy and independent aging, as illustrated by the 2020 AAL Program call for proposals (p. 5 [52]):

Proposed solutions should meet the needs of end-users, be it seniors, their carers, or institutions providing care. Innovative approaches to deployment and adoption of ICT services should be part of the solution development alongside the development of the new ICT/digital products, as well as their integration into the regional socio-economic context.

To reach the full potential of the ActiveAdvice platform, we should strive to improve the usability areas that we have highlighted in this study, namely efficacy and learnability. Simultaneously, to improve the chances that such a resource is recognized as useful by end users and its concepts are not totally unfamiliar, researchers and policy makers in the AAL field must determine how to better deliver information on AAL to those who need it. Pulling and retaining a user on the ActiveAdvice platform relies on 3 things: a *real need*, *knowing better* (ie, a perception that ICT-enabled solutions might help address an issue), and a *pleasant experience*. The former builds on the fact that the initial motivation for using such a platform rests on someone being at a point where they *need* advice regarding an actual (or predicted) loss of autonomy or quality of life. Getting the information out and disseminating the area both play a huge part in this, as people can learn that there might be something in the market that helps them to address a problem they might have that is affecting their independence and well-being. *Knowing better* can direct someone toward our solution when necessity occurs or is expected to occur. For a *pleasant experience*, being able to materialize that necessity into an answer is just as important. User-friendliness and simplicity are crucial when delivering a solution to someone who is often distrustful of information technologies, usually lacks the necessary patience to deal with them, and does not feel like thinking too much about things, frequently giving in to the *learned helplessness* phenomenon. To create products or services that are successful in the long run, it is necessary to ensure that the product has a sufficiently high engagement level for all relevant stakeholders, which is especially important for web services such as this platform. Making the developed concepts of ActiveAdvice not only useful for the (primary) end user but also supported and accepted by other stakeholders such as families, caregivers, product suppliers, and governments, is quite challenging but instrumental to achieving its full potential in supporting technology adoption by European older adults and, ultimately, healthy aging in place.

## Abbreviations

AAL: ambient assisted living
FCT: Fundação para a Ciência e a Tecnologia, IP
IC: informal carer
ICT: information and communications technology
PD: participatory design
SUS: System Usability Scale
UCD: user-centered design
